# Lack of Association of *C677T* Methylenetetrahydrofolate Reductase Polymorphism with Breast Cancer Risk in Mali

**DOI:** 10.1155/2023/4683831

**Published:** 2023-01-17

**Authors:** Brehima Diakite, Yaya Kassogue, Mamoudou Maiga, Guimogo Dolo, Oumar Kassogue, Jane L. Holl, Brian Joyce, Jun Wang, Kadidiatou Cisse, Fousseyni Diarra, Mamadou L. Keita, Cheick B. Traore, Bakarou Kamate, Sidi B. Sissoko, Bourama Coulibaly, Adama S. Sissoko, Drissa Traore, Fatoumata M. Sidibe, Sekou Bah, Ibrahim Teguete, Madani Ly, Sellama Nadifi, Hind Dehbi, Kyeezu Kim, Robert Murphy, Lifang Hou

**Affiliations:** ^1^Faculty of Medicine and Odontostomatology, University of Sciences, Techniques and Technologies of Bamako (USTTB), Bamako, Mali; ^2^Institute for Global Health, Northwestern University, Chicago, IL 60611, USA; ^3^Preventive Medicine Department, Northwestern University, Chicago, IL 60611, USA; ^4^Department of Neurology, University of Chicago, Chicago, IL 60637, USA; ^5^Faculty of Pharmacy, University of Sciences, Techniques and Technologies of Bamako (USTTB), Bamako, Mali; ^6^Hassan II University Aïn Chock, Casablanca, Morocco

## Abstract

Methylenetetrahydrofolate reductase (MTHFR) plays a major role in the metabolism of folates and homocysteine, which in turn can affect gene expression and ultimately promote the development of breast cancer. Thus, mutations in the *MTHFR* gene could influence homocysteine, methionine, and S-adenosylmethionine levels and, indirectly, nucleotide levels. Imbalance in methionine and S-adenosylmethionine synthesis affects protein synthesis and methylation. These changes, which affect gene expression, may ultimately promote the development of breast cancer. We therefore hypothesized that such mutations could also play an important role in the occurrence and pathogenesis of breast cancer in a Malian population. In this study, we used the PCR-RFLP technique to identify the different genotypic profiles of the *C677T MTHFR* polymorphism in 127 breast cancer women and 160 healthy controls. The genotypic distribution of the *C677T* polymorphism in breast cancer cases was 88.2% for CC, 11.0% for CT, and 0.8% for TT. Healthy controls showed a similar distribution with 90.6% for CC, 8.8% for CT, and 0.6% for TT. We found no statistical association between the *C677T* polymorphism and breast cancer risk for the codominant models CT and TT (*p* > 0.05). The same trend was observed when the analysis was extended to other genetic models, including dominant (*p* = 0.50), recessive (*p* = 0.87), and additive (*p* = 0.50) models. The *C677T* polymorphism of *MTHFR* gene did not influence the risk of breast cancer in the Malian samples.

## 1. Introduction

Breast cancer is a major public health problem in both developed and developing countries [1–3]. According to the Global Cancer Observatory on Cancer (GLOBOCAN 2020) report, breast cancer is the most common female cancer and the most frequently diagnosed cancer, followed by lung and prostate cancers, with 2.3 million new cases (11.7% of all cancers) and nearly 684,996 new deaths (6.9% of deaths of all registered cancers) [3]. Two recent reports (GLOBACAN 2018 and 2020) report that breast cancer ranks as the most common cancer among women followed by cervical cancer [2, 3]. The onset of breast cancer is a multifactorial process that involves clinical characteristics, lifestyle, environment, and genetic factors [4–6]. As a result, several mutations in certain genes are being investigated to better understand the mechanisms of breast cancer occurrence. However, it is important to emphasize that findings regarding involvement of these genes remain contradictory. Folates are involved in the process of carcinogenesis through the modulation of DNA methylation and the control of DNA synthesis based on daily food intake [7]. It should be noted that this contribution may vary depending on the polymorphism of the methylenetetrahydrofolate reductase (*MTHFR*) gene. *MTHFR,* known as an essential enzyme in folate metabolism, is involved in the regulation and conversion of homocysteine to methionine [8]. Indeed, *MTHFR* catalyzes the irreversible reduction of 5,10-methylenetetrahydrofolate (5,10-methylene-THF) to 5-methyl-THF. The major circulating form of folate, 5-methyl-THF, serves as a substrate for the methylation of homocysteine to methionine by using methionine synthase, which has vitamin B12 as a cofactor. Methionine enables the de novo biosynthesis of S-adenosylmethionine (SAM), which is the main donor of methyl radicals in humans [7, 8]. Many mutations have been identified in the *MTHFR* gene, including the substitution of a cytosine at position *677* by a thymine (*C677T*) [9, 10]. The distribution of this single nucleotide polymorphism (SNP) varies by study population and ethnic origins [11–15]. It has been reported that the TT mutant homozygote, which is associated with a significant decrease in the enzyme activity of *MTHFR*, is implicated in the processes of hypermethylation and hypomethylation of DNA [16] and could promote the development of certain cancers, such as breast cancer [14, 15, 17].

Data in the literature about the role of *C677T* in modulating the risk of cancer remain controversial. Several meta-analyses and reviews indicated that the *C677T* variant was associated with a risk of breast cancer [5, 15, 18, 19]. However, other reports have found no association [20, 21]. Given the impact of the *C677T* polymorphism on DNA methylation, we hypothesize that this polymorphism could play a major role in the pathogenesis of breast cancer in Mali, a multiethnic, West African population. To the best of our knowledge, data on the relationship between the *C677T* variant of *MTHFR* gene and the risk of breast cancer are lacking for the Malian population. In this study, we assess the association between the *C677T* polymorphism of *MTHFR* and breast cancer risk in a sample of Malian women with breast cancer and health controls.

## 2. Materials and Methods

### 2.1. Subjects

Study participants with breast cancer, who were seeking care at the oncology and pathology departments of University Hospital Center of Point G, Bamako, between July 2018 and January 2020, were recruited. Health controls were recruited at the Center National de Transfusion Sanguine (CNTS) in Bamako, Mali, which facilitated the recruitment of age-matched controls to patients. Age at diagnosis, tumor location, use of contraception, menopausal status, parity, breastfeeding, family history of breast cancer, history of benign breast disease, obesity, smoking, histological type, tumor size, lymph node involvement, and metastases, were manually extracted from each patient's operative record. Inclusion criteria of study participants with breast cancer were women of age 18–55 years with confirmed breast cancer, availability of demographic, clinical and histological information, and absence of other cancer(s) and/or chronic disease(s). Inclusion criteria for healthy control participants were women of age 18 to 55 years without any history of malignancy or chronic disease. The study was approved by the Ethics Committee of the Faculty of Medicine and Odontostomatology/Faculty of Pharmacy (2018/63/CE/FMPOS) at the University of Sciences, Techniques, and Technologies of Bamako (USTTB), and all participants provided informed consent before any study activities.

### 2.2. Genotyping of *MTHFR* C677T Polymorphism

**Figure 1:**
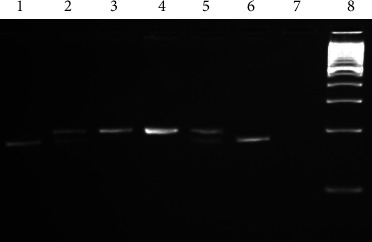
Genotypic profiles of the *C677T* polymorphism of *MTHFR* on agarose gel. Lane 8 represents DNA ladder; lanes 3 and 4 represent CC wild type; lanes 2 and 5 represent CT heterozygous; lanes 1 and 6 represent TT-mutated homozygous.

Genomic DNA was isolated from whole blood samples (5 ml of peripheral blood), using GentaPuregene Extraction Kit, Qiagen. Spectrophotometer was used to check the quantity and quality of the DNA. The PCR-RFLP technique was used to determine the *C677T* polymorphism of *MTHFR*, using the forward and reverse primers sequences, as described previously [9]. A 25 *μ*l final reaction volume containing 10 X reaction buffer, 25 mM MgCl_2_, 5 mM dNTP, 5 *μ*M primers, 500 U of Taq DNA polymerase, and 100 ng genomic DNA was used to amplify *C677T* from *MTHFR*. PCR amplification conditions included an initial denaturation step for 5 minutes (min) at 95°C, followed by 35 cycles: denaturation at 95°C for 1 min, annealing at 55°C for 1 min with extension at 72°C for 1 min. A final extension step was carried out at 72°C for 7 min. The PCR products were electrophoresed on 2% agarose gels stained with ethidium bromide. The C to T substitution at position 677 of *MTHFR* creates a restriction site with *HinfI* enzyme for the *C677T MTHFR* polymorphism. After amplification and digestion, PCR products showed one fragment of 198 bp for wild CC, three fragments of 198, 175, and 23 bp for CT and two fragments of 175 and 23 bp for TT (Figure 1). In addition, 10% of the sample was retested to ensure that the PCR results were accurate.

### 2.3. Statistical Analysis

SPSS 16.0 software (SPSS, Inc., Chicago, IL, USA) was used for statistical analysis. The relationship between the *C677T* polymorphism of *MTHFR* and demographic, clinical, and histological characteristics was assessed using the *χ*^2^ test. The same test was used to check Hardy−Weinberg equilibrium. The association between the *C677T* polymorphism and the risk of breast cancer in all genetic models (codominant: CT versus (vs.) CC and TT vs. CC, dominant: CT + TT vs. CC, recessive: TT vs CT + CC, additive: *T* vs *C*) was measured by calculating the OR with 95% confidence interval (CI). A *p* value less than 0.05 was considered significant in all tests.

## 3. Results

We recruited 127 patients with breast cancer, including 121 cases with invasive ductal carcinoma (IDC) and 6 cases with other histological forms (glycogen-rich clear cell carcinoma, lobular carcinoma in situ, moderately differentiated adenocarcinoma, and infiltrating adenocarcinoma) and 160 healthy controls. The mean age of patients was 43.30 ± 2.1 years and 41.2 ± 2.5 years for control subjects, respectively.  Table 1 shows the clinical and histological characteristics of the breast cancer group. Among breast cancer patients, 54.7% were ≤40 years old, 59.0% had a left breast tumor, 70.1% were not using a contraceptive, 52.8% were of childbearing age with 74.8% being nulliparous, 92.1% reported no family history of breast cancer, 67.7% were not obese, and 84.3% of patients were nonsmokers. Invasive ductal carcinoma was the most prevalent histological type (95.3%) compared to other types, including glycogen-rich clear cell carcinoma, lobular carcinoma in situ, moderately differentiated adenocarcinoma, and infiltrating adenocarcinoma. When considering the TNM classification (T, tumor size; N, nodal involvement; M, metastasis), T3, N0, and M0 were 63.0%, 56.7%, and 93.7%, respectively. No correlation was observed between the *C677T* polymorphism of *MTHFR* and clinicopathological characteristics in breast cancer patients (*p* > 0.05) (Table 1).

Table 2 represents the distribution of *C677T MTHFR* polymorphism the in breast cancer group according to genetic models. The distribution of genotypes was 88.2% for CC wild type, 11.0% for CT heterozygous, and 0.8% for TT mutant homozygous in the cases and 90.6% for CC, 8.8% for CT, and 0.6% for TT in the controls. The allelic frequencies in cases and controls were 93.7% for *C*, 6.3% for *T,* and 95.0% for *C*, 5.0% for *T*, respectively. None of the genetic models showed a significant association of the *C677T* polymorphism and risk of breast cancer, including the codominant model (CT vs. CC: OR = 1.29, 95% CI = 0.59–2.82 and TT vs. CC: 1.29, 95% CI = 0.08–20.92), dominant model (TT + CT vs. CC: OR = 1.29, 95% CI = 0.61–2.76; *p* = 0.50), recessive model (TT vs. CC + CT: OR = 1.26, 95% CI = 0.08–20.37; *p* = 0.87), and additive model (T vs. C: OR = 1.28, 95% CI = 0.62–2.61; *p* = 0.50) (Table 2). The distribution of genotypic and allelic frequencies was in agreement with Hardy−Weinberg equilibrium in both cases (*X*^2^ = 0.56, *p* = 0.45) and controls (*X*^2^ = 0.99, *p* = 0.32).

## 4. Discussion

We observed that the frequency of the mutant *T* allele in our healthy controls (5.0%) was statistically comparable to those reported in West African countries, including, Burkina Faso (6.0%) [22], Nigeria (6%) [23], Gambia (6.2%) [24], Togo (8.3%) [25], and Ghana (8%) [26]. In contrast, this frequency was lower than those observed in India (13.1%) [27], Morocco (24.0%) [14], Iran (21.7%) [21], Turkey (27.4%) [28], Brazil (31.2%) [29], United States of America (31.5%) [30], Italy (41.4%) [31], China (41.8%) [32], and Mexico (43.2%) [33]. The wide range distribution of the mutant *T* allele in healthy women appears to depend on the study population and ethnic origins.

The present case-control study revealed that the *C677T* polymorphism of *MTHFR* is not linked with the occurrence of breast cancer in a sample of women from Mali. Numerous studies have been undertaken to assess the influence of the *C677T* polymorphism of *MTHFR* in breast cancer, but the conclusions remain conflicted. Our results are consistent with previously published work, which found no significant association with risk of breast cancer [27–29, 31, 33]. Three recent meta-analyses, one comprising 75 studies with 31,315 cases and 35, 608 controls, the second comprising 39 studies with 19,260 cases and 26,364 controls, and the third comprising 67 studies with 23,440 cases and 27880 controls, showed no significant association between the *C677T* polymorphism of *MTHFR* and risk of breast cancer in Caucasian women, but not in Asian women [19, 20, 34] and mixed women population [34]. These results are in part consistent with the present study. Contrary to our findings, a meta-analysis comprising 9 studies with 2136 cases and 2436 controls showed a significant association of the *C677T* polymorphism with the risk of breast cancer in Latino women [35]. The discrepancy between studies of the *C677T* polymorphism of *MTHFR* with occurrence of breast cancer could be due to sample size, ethnic origins, genetic background, linkage disequilibrium, and in particular the lifestyle of the populations studied.

Although a clear mechanism by which the *MTHFR* mutation promotes the development of breast cancer has not yet been fully established, the *C677T* polymorphism has been considered to be a variant producing a thermolabile enzyme with limited activity. This variant is the most common genetic cause of hyperhomocysteinemia [36, 37]. In physiological situations, 5,10-methylene tetrahydrofolate reductase (*MTHFR*) catalyzes the irreversible reduction of 5,10-methylene tetrahydrofolate (*CH2THF*) to 5-methyltetrahydrofolate (*CH3THF*) [38]. Thus, the activity of *MTHFR* affects the availability of *CH2THF*, which influences the synthesis of RNA and DNA. *MTHFR* plays a major role in folate and homocysteine metabolisms by acting on the transfer of its methyl group during the remethylation of homocysteine by methionine synthase. *MTHFR* can modulate the levels of homocysteine, methionine, and S-adenosylmethionine and indirectly influence the levels of nucleotide, since its substrate, 5,10-methylene tetrahydrofolate, is used for the synthesis of thymidine [38]. The imbalance in methionine and S-adenosylmethionine synthesis affects protein synthesis or methylation reactions. Changes in DNA methylation affect gene expression, and *MTHFR* deficiency can promote the development of oncogenic processes [39, 40]. The redistribution of folate metabolites can affect the synthesis of purines and pyrimidines, which in turn affects DNA synthesis or repair. Supplementation or dietary intake of appropriate vitamins can alleviate potential *MTHFR* deficiencies. There is still a great deal of controversy about the role of *MTHFR* polymorphisms. However, a link with cardiovascular disease has been established, which may then have an indirect link with risk of breast cancer [41]. The *C677T* polymorphism of the *MTHFR* gene has been reported to be associated with an increased level of homocysteine, which may independently contribute to the development of stroke and coronary disease. The impact of the *C677T* polymorphism on the risk of developing of cardiovascular diseases, such as stroke (hemorrhagic or ischemic), venous thrombosis, and coronary heart disease has been reported in the Caucasian and Asian populations [41–44]. These observations confirm the fact that the impact of a SNP depends on the ethnic and geographical origin of the population studied.

This study has some limitations. First, the sample size is relatively small; therefore, the results cannot be generalized to the entire Malian population. Second, hormone receptor tests ((luminal A, luminal B, HER2 overexpression, and triple-negative subtypes) and blood folate levels could not be performed in the majority of patients. Determination of hormonal status enables better patient management while improving the prognosis. In addition, low blood folate levels may cause certain abnormalities such as cardiovascular and cancer diseases.

## 5. Conclusions

In conclusion, this study did not show any association between the *C677T* polymorphism of the *MTHFR* gene and risk of breast cancer. The distribution of the mutant T allele was very rare in both groups of women with and without breast cancer. Further studies with a folic acid assay should be performed to evaluate the association of the *C677T MTHFR* polymorphism and risk of breast cancer.

## Figures and Tables

**Table 1:** Distribution of *C677T MTHFR* polymorphism by clinicopathological characteristics in the breast cancer group.

Clinical parameter	*C677T MTHFR*					
	*N* = 127 (%)	CC	CT	TT	*X* ^2^	*p*
Age of diagnosis					1.45	0.48
≤40 years	69 (54.7)	59 (85.5)	9 (13.0)	1 (1.4)		
>40 years	57 (45.2)	52 (91.2)	5 (8.8)	—		
Localization of tumor					2.63	0.62
Right breast	43 (33.9)	36 (83.7)	6 (14.0)	1 (2.3)		
Left breast	75 (59.0)	68 (90.7)	7 (9.3)	—		
Bilateral	9 (7.1)	8 (88.9)	1 (11.1)	—		
Use of contraceptives				2.83	0.24	
No	89 (70.1)	78 (87.6)	11 (12.4)	—		
Yes	38 (29.9)	34 (89.5)	3 (7.9)	1 (2.6)		
Menopausal status					7.22	0.12
Premenopausal	17 (13.4)	14 (82.4)	2 (11.8)	1 (5.9)		
Postmenopausal	43 (33.8)	37 (86.0)	6 (14.0)	—		
Fertile women	67 (52.8)	61 (91.0)	6 (9.0)	—		
Parity					7.98	0.14
Nulliparity	15 (11.8)	13 (86.7)	2 (13.3)	—		
Primiparity	17 (13.4)	15 (88.2)	1 (5.9)	1 (5.9)		
Multiparity	95 (74.8)	84 (88.4)	11 (11.6)	—		
Family history of BC					0.10	0.95
Yes	10 (7.9)	9 (90.0)	1 (10.0)	—		
No	117 (92.1)	103 (88.0)	13 (11.1)	1 (0.9)		
Obesity					1.25	0.53
Yes	41 (32.3)	35 (85.4)	6 (14.6)	—		
No	86 (67.7)	77 (89.5)	8 (9.3)	1 (1.2)		
Smoking					2.10	0.35
Passive smoking	20 (15.7)	16 (80.0)	4 (20.0)	—		
No	107 (84.3)	96 (89.7)	10 (9.3)	1 (0.9)		
Histologic type					0.84	0.65
IDC	121 (95.3)	106 (87.6)	14 (11.6)	1 (0.8)		
Others	6 (4.7)	6 (100.0)	—	—		
Tumor size					3.92	0.68
T1	2 (1.6)	2 (100.0)	—	—		
T2	28 (22.0)	25 (89.3)	3 (10.7)	—		
T3	80 (63.0)	72 (90.0)	7 (8.8)	1 (1.3)		
T4	17 (13.4)	13 (76.5)	4 (23.5)	—		
Nodal involvement						
N0	72 (56.7)	65 (90.3)	7 (9.7)	—	4.24	0.64
N1	39 (30.7)	33 (84.6)	5 (12.8)	1 (2.6)		
N2	13 (10.2)	12 (92.3)	1 (7.7)	—		
N3	3 (2.4)	2 (66.7)	1 (33.3)	—		
Metastasis					0.08	0.96
M0	119 (93.7)	105 (88.2)	13 (10.9)	1 (0.8)		
M1	8 (6.3)	7 (87.5)	1 (12.5)	—		

IDC, invasive ductal carcinoma; *X*^2^, chi-squared test; *P* = *p* value; *N*, number; BC, breast cancer; CC, wild type; CT, heterozygous; TT, mutant homozygous. Others: glycogen-rich clear cell carcinoma, lobular carcinoma in situ, moderately differentiated adenocarcinoma, and infiltrating adenocarcinoma.

**Table 2:** Distribution of *C677T MTHFR* polymorphism in the breast cancer group according to genetic models.

Genotype/allele	Cases	Controls	OR (95% CI)	*p*
	*N* = 127	*N* = 160		
CC	112 (88.2)	145 (90.6)	Reference	
CT	14 (11.0)	14 (8.8)	1.29 (0.59–2.82)	0.52
TT	1 (0.8)	1 (0.6)	1.29 (0.08–20.92)	0.85
TT + CT	15 (11.8)	15 (10.0)	1.29 (0.61–2.76)	0.50
CC + CT	126 (99.2)	159 (99.4)	Reference	
TT	1 (0.8)	1 (0.6)	1.26 (0.08–20.37)	0.87
*C*	238 (93.7)	304 (95.0)	Reference	
*T*	16 (6.3)	16 (5.0)	1.28 (0.62–2.61)	0.50

*N*= number; CI: confidence interval; *p*: *p* value; TT + CT vs. CC: the dominant model; TT vs. CC + CT: the recessive model; *T* vs. *C*: the additive model;

## Data Availability

The datasets generated and/or analyzed in this study are available from the corresponding author upon request and with the permission of FMPOS/USTTB Ethics Committee.

## Abbreviations

CHU: University Hospital Center
CI: Confidence interval
HWE: Hardy–Weinberg equilibrium
LMICs: Low- and middle-income countries
MTHFR: Methylenetetrahydrofolate reductase
OR: Odds ratio
USTTB: University of Sciences, Techniques, and Technologies of Bamako.
